# An inductive exploration of the implementation knowledge of research funders

**DOI:** 10.1186/s12961-019-0472-8

**Published:** 2019-07-18

**Authors:** Anders Brantnell, Enrico Baraldi, Theo van Achterberg

**Affiliations:** 1 Department of Women’s and Children’s Health, Uppsala University, Uppsala, Sweden; 2 Department of Industrial Engineering and Management, Uppsala University, Uppsala, Sweden; 3 KU Leuven Department of Public Health and Primary Care, Academic Centre for Nursing and Midwifery, KU Leuven, Leuven, Belgium; 4 Radboud Institute for Health Sciences, Scientific Center for Quality of Healthcare, Radboud University Medical Centre, Nijmegen, The Netherlands

**Keywords:** Research policy, implementation, quality improvement, healthcare research, research funder, policy-maker, knowledge use

## Abstract

**Background:**

Healthcare research funders may undertake various roles to facilitate implementation of research findings. Their ability to enact such roles depends on several factors, knowledge of implementation being one essential requirement. However, previous studies do not assess the type or level of knowledge about implementation that research funders possess. This paper therefore presents findings from a qualitative, inductive study of the implementation knowledge of research funders. Three aspects of this knowledge are explored, namely how research funders define implementation, their level of self-assessed implementation knowledge and the factors influencing their self-assessment of implementation knowledge.

**Methods:**

Research funders (*n* = 18) were purposefully selected from a sample of research funding organisations in Sweden (*n* = 10). In-depth semi-structured interviews were conducted, recorded and transcribed verbatim. An inductive method using a systematic coding procedure was employed to derive the findings.

**Results:**

The research funders defined implementation as either an outcome or a process, with the majority believing that implementation of healthcare research results demands a process, although its complexity varied in the research funders’ view. They perceived their own level of implementation knowledge as either limited or substantial, with a majority regarding it as limited. Clinical research experience, clinical experience and task relevance were singled out as the clearest factors affecting the self-assessment of their own implementation knowledge.

**Conclusions:**

This study, the first to focus on implementation knowledge of research funders, demonstrates that they are a category of policy-makers who may possess knowledge, based on their previous professional experience, that is comparable to some important findings from implementation research. Consequently, the findings not only pinpoint the relevance of professional experience, but also reveal a lack of awareness and knowledge of the results of implementation research among research funders in charge of healthcare research.

**Electronic supplementary material:**

The online version of this article (10.1186/s12961-019-0472-8) contains supplementary material, which is available to authorized users.

## Background

Research funders and governments invest heavily in healthcare research. For instance, the European Union invested approximately EUR 6 billion in the ‘health’ theme of the Seventh Framework Programme [1], and the United Kingdom government alone allocates GBP 1.2 billion to healthcare research annually [2]. However, the resulting improvement in public health does not match the scale of investments, which suggests the existence of a knowledge–practice gap [3–6], where existing treatments are insufficiently based on available recommendations for best practice [7]. This, in turn, implies that patients receive unnecessary, too little or too much care [8], and that resources are used sub-optimally [5, 9]. Hulscher et al. [10] reported that, in 50% of cases, antibiotics are prescribed when they are unnecessary. Grol [11] stated that roughly 30% of patients in the Netherlands do not receive the recommended care. Berwick and Hackbarth [12] confirmed that overtreatment, such as performing surgery when waiting is recommended, is highly prevalent in the United States.

The need to address the knowledge–practice gap has stimulated the growth of implementation research, which is the scientific study of methods that support systematic introduction of research evidence into clinical practice with the aim of improving healthcare quality [13]. Implementation of new research evidence in clinical practice requires, first, identification of factors (e.g. lack of knowledge or awareness) that contribute to the behaviour observed (e.g. not adhering to existing guidelines) and, second, specific strategies (e.g. raising consciousness) to change the factors identified [14]. Consequently, since implementation requires behavioural change, it is highly complicated [14]. Recently, governments have acknowledged and emphasised the knowledge–practice gap [15, 16]. One proposed solution to diminish this gap has been to expand the roles of healthcare research funders beyond their traditional roles of evaluating and funding grant proposals [16]. Consequently, the strategic position of research funders, operating between healthcare research and healthcare practice, has been acknowledged [17].

Previous studies have identified several facilitative roles for research funders before, during and after implementation [18–22]. Before implementation, one problem arises if the research conducted fails to match health professionals’ needs [23, 24]. To address this issue, research funders have encouraged and established links between researchers and health professionals [22, 25] with a view to enhancing scope for acceptance and implementation. Further, research funders can also impact research agenda-setting by allocating resources to implementation research or, alternatively, inducing researchers to consider or prepare for implementation in their grant applications [15, 26].

During implementation, a lack of resources to implement new evidence is another problem [27, 28]. To address this, the research funders’ role can be to provide funds earmarked for supporting the implementation process [15, 29]. Finally, a key problem associated with implementation is adherence to the new practice. In general, only a 10% change in behaviour may be expected as a result of implementation efforts [30]. Where adherence to new practices is low, research funders can adopt post-implementation roles such as following up implementation to evaluate how far their investments actually improve care [20]. Accordingly, research funders execute policies at the research funding organisations and can perform various roles before, during and after implementation, thereby helping to diminish the knowledge–practice gap.

Despite evidence supporting various facilitative roles for research funders, they do not, in general, adopt roles that go beyond evaluating and funding proposals [31]. To understand the preconditions for their actual performance of facilitative roles, one needs to consider the factors that influence the behaviour of research funders. Relevant factors include their knowledge, beliefs, attitudes, values and expectations. Although all these factors are important, knowledge is a precondition for many others [32]. Without knowledge, some actors might not have developed beliefs, for example. Other actors might have developed some beliefs but remained unable to fully consider a specific concept, such as implementation, and develop or test their own understanding or judgment of it [32]. However, despite the importance of research funders’ knowledge about implementation in framing their facilitative roles, studies focusing explicitly on such knowledge are lacking.

Knowledge may relate to understanding of a concept [32], such as various ways to define ‘implementation’, or include the level of a research funder’s knowledge captured through self-assessment [33]. Knowledge can also be variously categorised, but a general distinction is made between experience-based knowledge [34–37] and science-based knowledge [3, 38–40]. Concerning the latter, implementation research has generated a large scientific output that can be used to plan, conduct and evaluate implementation efforts [41]. Further, two general insights from implementation research are that implementation is a complex process [6, 38, 42–44] and that it requires a strategy for identifying and addressing barriers to and facilitators of implementation [28, 39, 45–47]. However, whether these scientific results and this kind of knowledge are used by research funders – who are one type of policy-makers – to guide their possible facilitative roles is unclear. Although studies addressing research funders specifically are lacking, studies on policy-making in general suggest that policy-makers seldom rely on science-based recommendations. This implies the existence of a knowledge–policy gap [48–52].

On the other hand, research funders are a special type of policy-maker because they act between healthcare research and healthcare practice. To this end, research funders may have acquired experience-based implementation knowledge. Models and frameworks from policy and implementation literature focusing on science-based knowledge [43, 47, 53, 54] may thus not suffice to explain these managers’ implementation knowledge. Factors that can impact experience-based knowledge are work experience and educational or research background [34, 36, 55]. For instance, research funders may have work experience from clinical (i.e. healthcare) and industrial (e.g. pharmaceutical) settings as well as practical experience from either clinical (i.e. patient related) or general research areas (e.g. biology or chemistry).

Against this background, the purpose of this paper is to inductively develop a model that can explain research funders’ implementation knowledge and its origins. The research questions addressed in this paper are as follows: (1) How do these research funders define ‘implementation’? (2) What level of self-assessed implementation knowledge do they possess? and (3) What factors influence their self-assessed implementation knowledge?

## Methods

### Study design

A multiple inductive case study, involving purposefully selected research funders in Sweden, was conducted. The aim was to develop a model, based on case-study observations, by comparing similarities and differences among the cases selected [56–58] concerning implementation knowledge. We found that the literature on implementation and policy-making focused predominantly on science-based knowledge, but that research funders work in the interface between healthcare practice and healthcare research, suggesting that the focus on science-based knowledge is inadequate. Consequently, we chose an inductive approach [58] to collect data on implementation-related knowledge in general, without applying predefined categories or theoretical models. Only after data collection did we compare our findings with those in the academic literature. The units of analysis were the research funders, who are the key decision-makers at each funding organisation. Semi-structured interviews were first conducted with the research funders. Then, to evaluate the consistency of the findings, secondary data were collected from the research funders’ institutional homepages [59]. This study is reported in accordance with Consolidated Criteria on Reporting Qualitative Research, COREQ [60].

### Case selection and respondent criteria

The leading principle for sampling the funders (*n* = 10) was to create variation in two dimensions, namely regarding closeness to implementation contexts and type of research funded (i.e. basic research, clinical research or a combination of the two). The research funders working in funding organisations operating closer to implementation contexts were assumed to have acquired implementation knowledge through their clinical work, whereas those far from implementation contexts were assumed to lack such knowledge. Similarly, research funders working at organisations supporting clinical research were assumed to have acquired implementation knowledge through their experience from clinical research, whereas those who fund basic research were assumed to lack experience-based implementation knowledge. This assumption led us to distinguish between three types of funding organisations, which were labelled as follows: (1) ‘FarBas’ (farthest from implementation, since these organisations belong to the apparatus of central government in Sweden and primarily fund basic research); (2) ‘CloserBoth’ (closer to implementation, since these funders, typically private foundations, operate in closer contact with specific clinical fields and fund both basic and clinical research); and (3) ‘ClosestClin’ (closest to implementation, since these funders belong to the organisations that provide healthcare in Sweden and primarily fund clinical research). Table 1 below provides details of the two sets of sampling criteria.

**Table 1:** Sampling criteria

Funders	Labelled	Areas of research supported	Closeness to implementation context
Funders 1–3	FarBas	Primarily basic research	Not close
Funders 4–6	CloserBoth	Combination of basic and clinical research	Closer
Funders 7–10	ClosestClin	Primarily clinical research	Closest

Our units of analysis were individuals, i.e. the research funders (*n* = 18), who were in turn selected to represent the key decision-makers at each funding organisation in terms of allocation of funds. They held such positions as chairman, vice chairman and general director. All the research funders approached agreed to participate in the study. To capture possible variation among individuals working within the same organisation, we selected two research funders from each funding organisation, except for two funding organisations where only one key decision-maker qualified as a respondent, based on the above criteria of seniority and decision-making power. We summarised each interview through field notes immediately after conducting it but we conducted no coding at this stage. We noticed that we reached data saturation, concerning implementation knowledge, after 10 interviews and considered that the initially included 10 research funding organisations constituted an adequate sample. However, to capture possible variation among funders in terms of our two selection criteria of implementation closeness and the type of research funded, we proceeded to interview the remaining eight respondents.

### Data collection

In-depth semi-structured interviews were conducted with the research funders to explore their implementation knowledge. In assessing this knowledge, we focused on three aspects. The first was how the research funders defined ‘implementation’, because we deemed that this might capture their basic understanding of implementation. The second aspect was their self-assessment of their own implementation knowledge because we were interested in exploring its level. Third, we focused on the factors influencing self-assessed implementation knowledge. Initially, we were also interested in covering the factors that influenced their implementation definitions, but noticed that the research funders provided explanations only of their self-assessed implementation knowledge and not of how they defined ‘implementation’. We therefore decided to focus only on factors that influenced self-assessed implementation knowledge. To capture the respondents’ own knowledge and interpretations, we probed their definitions of ‘implementation’ and self-assessment of implementation knowledge without explaining or clarifying to them what we meant by ‘implementation’.

One researcher (AB) approached the research funders through regular mail. The background of the study was outlined, brief reasons for the research funders’ participation were provided, and details of how data would be stored and handled were given. The letters were followed by phone calls to ask the research funders to participate in the study and answer their questions about the study, if any. We explained to the participants that accepting our request for the interview equated to providing consent to participate in the study. However, we underlined that they could withdraw from the study at any time without specifying a reason.

All the interviews were conducted by AB, who had in-depth knowledge of interview methods and qualitative research. AB did not know any of the research funders before conducting the interviews. Most of the interviews were conducted at the research funders’ offices (*n* = 17) and only one took place at the researcher’s university premises. An interview guide was used and adapted to different funders. Among the issues explored were how the research funders defined ‘implementation’ and their self-assessment of implementation knowledge. Prompts were given and clarifying questions asked where necessary. Only the researcher and the respondent were present when the interviews were conducted. The interviews were conducted face to face, and lasted 30–90 min. They were recorded and transcribed verbatim, and took place between April and September 2012. The interviews were conducted in Swedish and transcribed in the same language. The interview transcripts were translated to English during data analysis and the translations were checked by all authors to increase consistency and authenticity.

We also collected secondary data from the research funders’ institutional homepages to cover the professional background factors influencing research funders’ self-assessed implementation knowledge. The pages were searched for the professional background factors identified in the interview data (such as clinical experience), and the outcomes were measured dichotomously (‘Yes’ or ‘No’). If a specific professional background factor was presented on the page this was coded as ‘Yes’, and otherwise it was coded as ‘No’. The binary classification was based on the assumption that if a specific professional background factor is possessed by a research funder it would be presented on the homepage; clinical research experience, for example, is a qualification. Table 3 in the Results section shows the secondary data collected, along with the self-assessed implementation knowledge. One researcher (AB) collected the data from the research funders’ homepages. Collection of the secondary data took place in November 2015. In this way, triangulation was used to enhance the consistency of the findings [59].

### Data analysis

Adhering to an inductive approach, theory development being the goal, we applied a systematic coding procedure followed by a structured presentation of the data, resulting in a grounded theory [57]. The analysis was divided into six distinct phases. First, the transcripts were analysed and first-order categories were identified to reflect the specific implementation definitions and levels of self-assessed implementation knowledge, as perceived by the respondents. Second, the first-order categories were grouped into second-order themes to shift the interpretation of knowledge toward more abstract concepts. To achieve this, we compared the first-order categories with existing research on implementation and defined second-order themes that more closely reflected the implementation definitions in the existing literature. We were unable to find any existing research on research funders’ level of implementation knowledge, and the second-order themes concerning self-assessment were therefore formed without inputs from existing research.

Third, the second-order themes were grouped into higher-order aggregate dimensions, which described these [56, 57] based on previous research on implementation. These concerned implementation definitions but not self-assessment. The coding in phases 1–3 was initially conducted by AB, and discussed extensively by all the authors (AB, EB, TvA), whereupon some of the codes were changed and refined. The three researchers provided a good mix of different backgrounds (AB being a policy researcher, EB a management researcher and TvA an implementation researcher, health scientist and nurse), which forced us to reflect on the impact of our backgrounds on every phase of the research process [61]. To test the validity of the coding, we also asked four independent researchers to combine the first-order categories with the corresponding second-order themes, and the second-order themes with the corresponding aggregate dimensions. Based on their work, we refined the coding to enhance consistency. In detail, each code (24 in total for first-order categories, second-order themes and aggregate dimensions) was graded and each code (12 in total) that had less than 75% convergence among the four raters was reassessed. The reassessment of codes was discussed by the three authors and the final coding was based on a consensus among us. This recoding brought about no crucial change in the findings but merely improved the consistency of the coding throughout.

The fourth step in the analysis consisted of comparing the implementation definitions, self-assessed implementation knowledge and factors influencing the self-assessed implementation knowledge, within and among the three different types of funders (Table 1). These types were selected to provide variation in closeness to implementation and the type of research funded. Fifth, explanations for differences and similarities among research funders were explored on the basis of the interview data, which were also compared with the secondary data from the homepages. The latter supported the findings from the interviews. If a research funders’ self-assessed implementation knowledge was limited because of a lack of clinical experience, for example, the secondary data indeed confirmed that the manager lacked clinical experience. Conversely, if a manager had substantial self-assessed implementation knowledge and also mentioned the influence of clinical research experience and clinical experience, the secondary data confirmed that the manager had both these types of experience.

Sixth, the last stage in the analysis was the drafting of the grounded model. In developing this model and assessing the relevance of the professional background factors influencing self-assessed implementation knowledge, we combined the evidence for the factors from both the interview data and the secondary data. This integrated evidence from the two data sources allowed us to assess the relevance of each factor related to professional background. Based on this, our empirical findings (i.e. interview and secondary data) and the existing literature (e.g. policy research and implementation research) were compared to link the grounded model with existing research and thus provide a more robust grounded model. To capture existing research, a literature review was carried out. Its key findings are cited in the discussion section, where the grounded model is discussed in the light of the existing literature.

Ethical approval was applied for, but the Regional Ethical Review Board in Uppsala, Sweden, stated that no ethical approval was required for the study under Swedish legislation. In terms of ethical aspects, all the respondents gave their verbal consent to participate in the study when the invitation to participate in the study was followed up by telephone. During these telephone calls, AB proposed a time for an interview. It was explained that, if the respondent agreed to be interviewed, we considered this proof of explicit and valid verbal consent. Written consent was not requested, for two reasons. First, the respondents were in general extremely busy people, difficult to get hold of. Second, we did not wish to bother them with a written consent form when this is not, in fact, required by Swedish law [62]. Some of the respondents said that they had only half an hour for the interview, and if we had asked them to read and fill out a written consent form this would have reduced the interview time. The respondents’ consent to participate was documented through recorded and transcribed interviews following their approval. The whole process of obtaining verbal consent was described in detail in the application submitted to the Regional Ethical Review Board in Uppsala.

## Results

### Research funders’ definitions of ‘implementation’

**Fig. 1:**
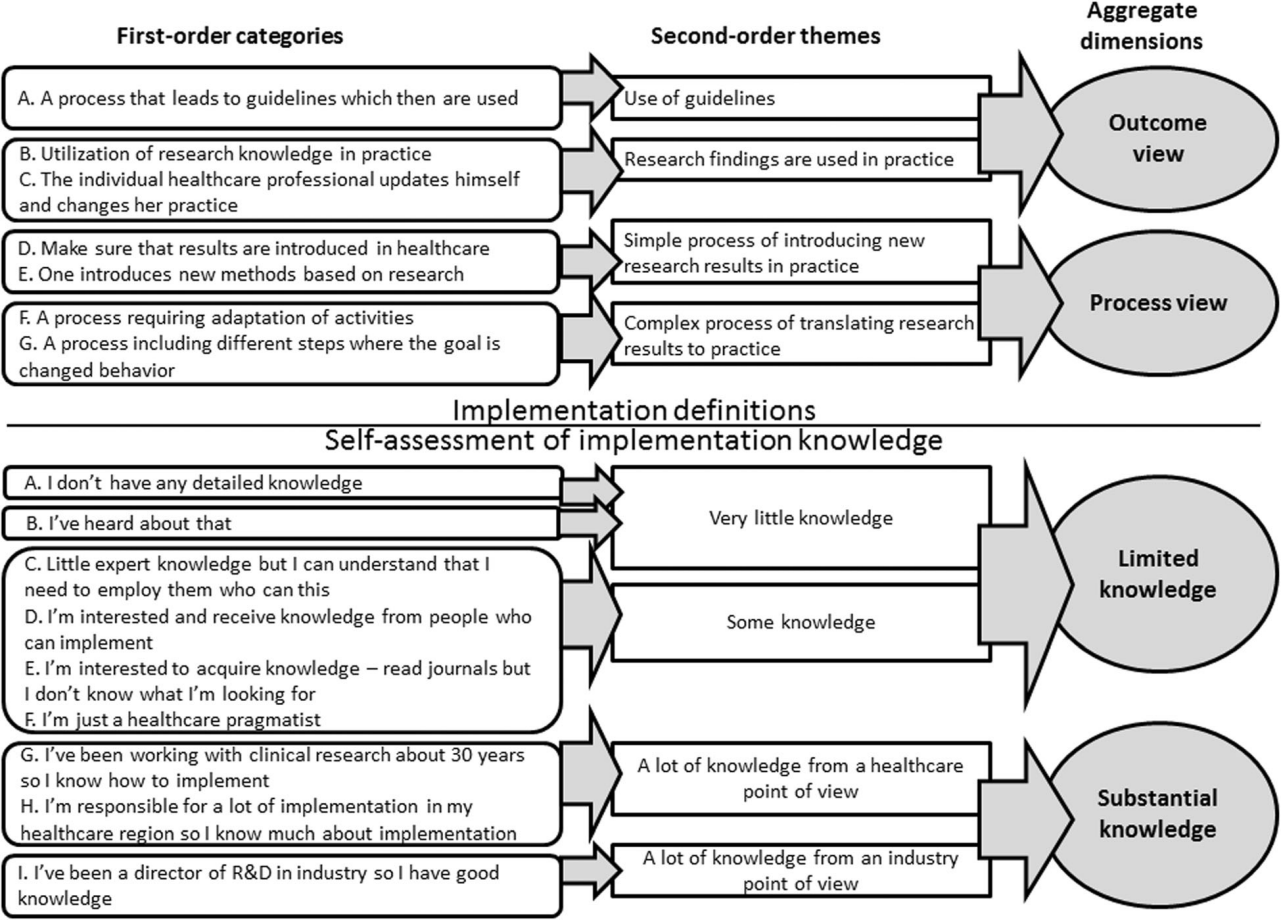
Data structure. Data structure describes the first three steps in data analysis where first-order categories, second-order themes and aggregate dimensions are formed concerning implementation definitions and self-assessment of implementation knowledge. The aggregate dimensions concerning implementation definitions were ‘outcome view’ and ‘process view’. The aggregate dimensions concerning self-assessment of implementation knowledge were ‘limited knowledge’ and ‘substantial knowledge’

We divided the implementation definitions provided by the research funders into two clearly different aggregated dimensions, namely ‘outcome view’ and ‘process view’. Each research funder defined ‘implementation’ as either an outcome or a process (Fig. 1). The ‘process view’ contained two second-order themes reflecting the different levels of complexity that research funders attributed to the implementation process; these were ‘simple process of introducing new research results in practice’ and ‘complex process of translating research results to practice’. The ‘outcome view’ also comprised two second-order themes, namely ‘use of guidelines’ and ‘research findings are used in practice’. Below, the different implementation definitions are elucidated in detail, supported by quotations from the interviews (Additional file 1 provides a structured overview of the implementation definitions, based on quotations).

### Definition of ‘implementation’ as an outcome

The ‘outcome view’ was based on two distinct second-order themes. The first focused on use of guidelines and the second on use of research findings in general. Admittedly, guidelines are research results too, but we made a distinction between the two themes, where the first second-order theme (‘use of guidelines’) depicted the guidelines as something that automatically inspires a wish to use the guidelines in practice:

“[Y]*ou then get these results applied and that you follow up their application and you write guidelines. Yes, when one has written the guidelines some people at the clinic become responsible and they will follow up guidelines. You can’t do anything else. I mean, today we work on the basis of guidelines, so everybody knows how to work with the guidelines*.” (Respondent 1 – CloserBoth Funder 5)

In this view, the existence of the guidelines is both a sufficient and a necessary condition for implementation or, in other words, nothing else is needed or can be done to obtain implementation. Similarly, the other second-order theme (‘research findings are used in practice’) indicated that these research funders perceived implementation as a state rather than a process, explaining it as something that plainly takes place when new research findings are available. Implementation was mentioned with no reference to third-party actions being necessary, and a processual element of implementation was thus lacking:

“*I define it as the new research findings being used – utilised and used in practice*.” (Respondent 2 – FarBas Funder 1)

### Definition of ‘implementation’ as a process

The ‘process view’ was divided into two distinct second-order themes (Fig. 1). The first, the ‘simple process of introducing new research results in practice’, focused on introduction of research results. Although this description does not characterise the process itself, this view nonetheless qualified as a ‘process view’ because implementation was perceived as something that required action:

“[M]*ake sure that a product or a service or a process starts and works in real life.*” (Respondent 1 – FarBas Funder 1)

This simple ‘process view’ may be contrasted with a complex ‘process view’ of the other second-order theme (‘complex process of translating research results to practice’), where one part contained the respondents’ description of the nature of the implementation process and the second part extended to their recognition of the end goal of implementation, i.e. modified behaviour. The first part of the complex ‘process view’, focusing on describing the process, stressed that adaptation of activities is the key issue in implementation:

“*It is completely illogical. You can’t foresee it. There are some general steps. You need to be flexible – to be able to adjust, you need to have a plan, you need to have the right people on board, and you need to know which steps you need to go through. And then you need to have an adaptive project plan that can be adjusted, depending on the reality you find when you approach the goal*.” (Respondent 1 – FarBas Funder 3)

The second part of the complex ‘process view’ acknowledged that implementation requires behavioural change:

“[T]*he goal of implementation is changed behaviour so that one gets another outcome for the customer – the patient. And it can be more or less difficult, depending on what’s going to be implemented … And then it has a lot to do with education – motivating, setting clear goals, arranging activities, carrying them out, following up and evaluating. So it’s sort of like that: a lot of support is often needed*.” (Respondent 2 – ClosestClin Funder 8)

Consequently, a common feature of the ‘process view’ was the view that implementation requires concerted efforts to ensure that research results are implemented. A common feature of the ‘outcome view’, on the other hand, was the perception of implementation as something that just happens. Overall, most of the research funders adhered to a ‘process view’. In terms of funder types, all three funding levels generally expressed a process view, except for the funders labelled as ‘CloserBoth’ (closer to implementation and funding both basic and clinical research), half of whose research funders expressed a process view and the other half an outcome view.

### Research funders’ self-assessed implementation knowledge

We divided the research funders’ self-assessed implementation knowledge into two aggregated dimensions, namely ‘limited knowledge’ and ‘substantial knowledge’. ‘Limited knowledge’ consisted of two distinct second-order themes, ‘very little knowledge’ and ‘some knowledge’, reflecting different degrees of ‘limited knowledge’. The ‘substantial knowledge’ dimension also contained two second-order themes, ‘a lot of knowledge from a healthcare point of view’ and ‘a lot of knowledge from an industry point of view’, reflecting different facets of ‘substantial knowledge’. Below, these different degrees and facets of the research funders’ knowledge about implementation are clarified (Additional file 1 provides a structured overview of their self-assessed knowledge, based on quotations).

### Implementation knowledge self-assessed as limited

‘Limited knowledge’ was based on two distinct second-order themes, depending on the degree of knowledge – ‘very little knowledge’ and ‘some knowledge’. There was also some variation within the theme ‘very little knowledge’. Some research funders considered that they had no detailed knowledge acquired through practice:

“*Not much at all. You mean in healthcare? No*.” (Respondent 1 – ClosestClin Funder 8)

Others stated that they had heard about implementation, but had very rudimentary knowledge:

“*That* [knowledge] *is very rudimentary. I’m an experimental* [researcher] *person*.” (Respondent 1 – CloserBoth Funder 6)

Respondents claiming ‘very little knowledge’ had in common a view of implementation as an issue separate from the research funder’s work, and they described it as something that they did not have to know about. Further, lack of clinical experience was associated with ‘very little knowledge’. They made references to their profession as researchers, which was used to justify their lack of knowledge about implementation, reinforcing an idea that research and implementation of research results are in fact separate activities. There was also variation in descriptions concerning ‘some knowledge’. Some respondents perceived that they had no expert knowledge, but understood enough to be able to identify the right people, with extensive knowledge about implementation:

“*Very modest, I mean very little expert knowledge, which doesn’t bother me at all, but I can understand the value of implementation and understand, when we talk about implementation, that I need to employ those who care about this* [implementation]. *It* [possessing implementation knowledge] *is kind of not my job*.” (Respondent 2 – CloserBoth Funder 4)

Another group of research funders considered that they had acquired some knowledge, either in interaction with implementation practitioners (i.e. industry representatives) or through self-education in implementation research (i.e. reading literature). For instance, interaction with industry was perceived to have contributed to their implementation knowledge:

“*Too little, I dare to say … what is still most exciting is when we talk to different companies that are trying to implement new drugs, new methods and similar things. We talk a lot about that, which is exciting and interesting. I get more of this kind of knowledge from them than I get from the county council’s own healthcare organisation*.” (Respondent 2 – ClosestClin Funder 9)

One research funder who did not perceive that she had expert knowledge was nevertheless interested in acquiring knowledge of implementation research:

“*I can’t say that I have any specific knowledge … I’m interested in acquiring knowledge. I read journals but I don’t know what I’m looking for*.” (Respondent 2 – FarBas Funder 1)

Finally, the last group of research funders assigned to the second-order theme of ‘some knowledge’ had experience from healthcare, either as medical practitioners or as pharmaceutical industry representatives. For instance, one research funder perceived that implementation was part of the medical practitioner’s daily work and thus implied that all medical practitioners have some knowledge of implementation:

“[I have] *layman knowledge and acknowledge that we need to absorb and implement. It is part of the physician’s job, in my opinion … There’s not one thing that’s the same. I mean, this is a weird question for us doctors because we need to change all the time*.” (Respondent 1 – ClosestClin Funder 7)

To sum up, among the respondents with self-assessed implementation knowledge in the ‘some knowledge’ category, there were those who considered that possessing implementation knowledge was not their responsibility (i.e. ‘not my task’), others who perceived that they ought to have implementation knowledge and employed different strategies to acquire it (i.e. ‘my task’). and some who considered that they had received some knowledge through experience (i.e. clinical practice and industry experience) and knowledge of research (i.e. implementation research). In contrast, respondents in the second-order category of ‘very little knowledge’ had in common the fact that they did not view possessing implementation knowledge as relevant for research funders (i.e. ‘not my task’). Reasons for ‘very little knowledge’ were perceived as due either to the research funder being a researcher or to the research funder’s lack of clinical experience. Accordingly, the factors perceived by the research funders to influence their limited self-assessed implementation knowledge were (1) task relevance, (2) clinical experience, (3) industry experience, (4) knowledge of implementation research, and (5) general research experience.

### Implementation knowledge self-assessed as substantial

In contrast to ‘limited knowledge’, some research funders perceived that they had ‘substantial knowledge’, acquired through experience from either healthcare or industry. Consequently, the two second-order themes were ‘a lot of knowledge from a healthcare point of view’ and ‘a lot of knowledge from an industry point of view’. However, within the former second-order theme, ‘a lot of knowledge from a healthcare point of view’, there was variation in the types of experience the research funders had. The first group of research funders referred to clinical research:

“*You know, if you’ve been involved like I have, you get experience. I’ve been working in clinical research since 1970 so I know. Experience from these years gives knowledge, so to speak. Research results and how to implement them, what’s possible and what isn’t*.” (Respondent 1 – CloserBoth Funder 5)

The second group referred to clinical experience and responsibility for implementing research results:

“*Yes, a lot* [of knowledge]. *I’ve been a director of* [a clinical unit] *for many years and I’ve also been the director of* [a specialist medical research unit]*, so I have quite extensive experience of what it means, organisationally and from a resource point of view, when you change healthcare. Whether it’s a new method or a new drug, I have extensive experience of what that process is like*.” (Respondent 2 – CloserBoth Funder 5)

The other second-order theme of ‘substantial knowledge’ was ‘a lot of knowledge from an industry point of view’. In this case, implementation knowledge was acquired through extensive experience from industry, where the research funder had worked in research and development:

“*I’ve been a director of R&D at* [a large multinational company] *for* [several] *years, so I have good knowledge about that*.” (Respondent 1 – FarBas Funder 3)

The respondents perceived that ‘substantial knowledge’ originated from different types of experience (i.e. clinical experience, industry experience and clinical research experience). Moreover, none of these research funders stated that they had acquired their knowledge through the literature on implementation; rather, they stated explicitly that professional experience affords knowledge. Overall, most of the research funders assessed their implementation knowledge as ‘limited’. In terms of funding levels, the FarBas funders and the ClosestClin funders generally expressed limited self-assessed implementation knowledge, whereas the CloserBoth funders were divided between limited and substantial self-assessed implementation knowledge (Table 1).

### Factors influencing research funders’ self-assessed implementation knowledge

The research funders mentioned six factors that influenced their self-assessed implementation knowledge – general research experience, clinical research experience, clinical experience, industry experience, knowledge of implementation research, and task relevance (Table 2). When these factors influencing self-assessment were compared across the three funding levels, three factors emerged that, according to the research funders, in general, were not particularly important in influencing their implementation knowledge (general research experience, industry experience and knowledge of implementation research). Two of the factors, clinical research experience and clinical experience, were acknowledged as important by the CloserBoth research funders but not the FarBas and ClosestClin research funders. Finally, the research funders from the three funding levels had different views about the importance of task relevance. FarBas research funders generally considered that possessing implementation knowledge was not their task, whereas CloserBoth research funders had the opposite view. ClosestClin research funders’ views displayed no clear pattern.

**Table 2:** Factors cited by research funders as influencing their self-assessed implementation knowledge

Funders	General research experience	Clinical research experience	Clinical experience	Industry experience	Knowledge of implementation research	Task relevance	Implementation definitions	Self-assessed implementation knowledge
Funder FarBas1	1: NM^a^	1: NM	1: NM	1: NM	1: NM	1: NT^f^	1: Process	1: Limited
	2: NM	2: NM	2: NM	2: NM	2: L	2: NT	2: Outcome	2: Limited
Funder FarBas2	1: N^b^	1: NM	1: NM	1: NM	1: NM	1: NT	1: Process	1: Limited
	2: NM	2: NM	2: Y	2: NM	2: NM	2: MT^g^	2: Process	2: Substantial
Funder FarBas3	1: Y^c^	1: NM	1: NM	1: Y	1: NM	1: NM	1: Process	1: Substantial
Funder CloserBoth4	1: NM	1: NM	1: NM	1: Y	1: L	1: NM	1: Outcome	1: Limited
	2: NM	2: NM	2: NM	2: NM	2: LK^e^	2: NT	2: Process	2: Limited
Funder CloserBoth5	1: NM	1: Y	1: Y	1: NM	1: NM	1: MT	1: Outcome	1: Substantial
	2: NM	2: Y	2: Y	2: NM	2: NM	2: MT	2: Process	2: Substantial
Funder CloserBoth6	1: N	1: NM	1: NM	1: NM	1: NM	1: NM	1: Process	1: Limited
	2: NM	2: Y	2: Y	2: NM	2: NM	2: MT	2: Outcome	2: Substantial
Funder ClosestClin7	1: NM	1: NM	1: Y	1: NM	1: NM	1: MT	1: Outcome	1: Limited
	2: NM	2: NM	2: NM	2: NM	2: NM	2: NM	2: Process	2: Limited
Funder ClosestClin8	1: NM	1: NM	1: L	1: NM	1: NM	1: NM	1: Process	1: Limited
	2: NM	2: NM	2: NM	2: NM	2: NM	2: NT	2: Process	2: Limited
Funder ClosestClin9	1: NM	1: NM	1: NM	1: NM	1: NM	1: NM	1: Outcome	1: Limited
	2: NM	2: NM	2: NM	2: NM	2: NM	2: MTI^h^	2: Process	2: Limited
Funder ClosestClin10	1: L^d^	1: NM	1: NM	1: NM	1: NM	1: MTI	1: Outcome	1: Limited

^a^NM indicates that the factor in question was not mentioned by the research funder

^b^N means that the factor applied to the respondent but the respondent considered that it impacted their self-assessed implementation knowledge negatively

^c^Y means that the factor applied to the respondent and that the respondent considered that it impacted their self-assessed implementation knowledge positively

^d^L means that the research funder (1) mentioned the factor, and (2) explicitly considered that their lack of this factor reduced their self-assessed implementation knowledge

^e^LK means that the research funder simply expressed that they did not possess the factor in question

^f^NT stands for ‘not my task’

^g^MT stands for ‘my task’

^h^MTI stands for ‘my task with an aim to increase my knowledge’

The two factors acknowledged as important by the CloserBoth research funders (clinical research experience and clinical experience) were, in general, connected to substantial self-assessed implementation knowledge, whereas their absence was connected to limited self-assessed implementation knowledge. Regarding task relevance, the FarBas research funders perceived possessing implementation knowledge as not being part of their task and had limited self-assessed implementation knowledge, whereas the opposite was true of the CloserBoth research funders. Table 2 also refers to implementation definitions and self-assessed implementation knowledge, showing that ClosestClin research funders perceive their own knowledge as limited but may nonetheless define implementation as either a process or an outcome. CloserBoth, on the other hand, includes many research funders who claim substantial implementation knowledge but may nonetheless perceive implementation as both a process and an outcome.

Moreover, we triangulated the four factors connected to the research funders’ professional background and observed that the interview findings were confirmed by the secondary data (Table 3). Here, too, the CloserBoth research funders stand out in terms of their clinical research experience and clinical experience, whereas industry experience is not prevalent among the three funding levels. Both the interview data and the secondary data indicate that the two factors ‘clinical research experience’ and ‘clinical experience’ were, in many cases, connected to substantial self-assessed implementation knowledge across the three funding levels, whereas their absence was connected to limited self-assessed implementation knowledge. Regarding general research experience, Table 3 shows that it is lacking from the majority of the ClosestClin research funders but possessed by the majority of research funders from the other two funding levels.

**Table 3:** Research funders’ professional background^a^ and self-assessed implementation knowledge

Funders	General research experience	Clinical research experience	Clinical experience	Industry experience	Self-assessed implementation knowledge
Funder FarBas 1	1: No	1: No	1: No	1: No	1: Limited
	2: No	2: No	2: No	2: No	2: Limited
Funder FarBas 2	1: Yes	1: No	1: No	1: No	1: Limited
	2: Yes	2: Yes	2: Yes	2: No	2: Substantial
Funder FarBas 3	1: Yes	1: No	1: No	1: Yes	1: Substantial
Funder CloserBoth 4	1: Yes	1: Yes	1: Yes	1: Yes	1: Limited
	2: No	2: No	2: No	2: No	2: Limited
Funder CloserBoth 5	1: Yes	1: Yes	1: Yes	1: No	1: Substantial
	2: Yes	2: Yes	2: Yes	2: No	2: Substantial
Funder CloserBoth 6	1: Yes	1: No	1: No	1: No	1: Limited
	2: Yes	2: Yes	2: Yes	2: No	2: Substantial
Funder ClosestClin 7	1: Yes	1: No	1: Yes	1: No	1: Limited
	2: Yes	2: Yes	2: Yes	2: No	2: Limited
Funder ClosestClin 8	1: Yes	1: No	1: No	1: No	1: Limited
	2: No	2: No	2: Yes	2: No	2: Limited
Funder ClosestClin 9	1: No	1: No	1: No	1: No	1: Limited
	2: No	2: No	2: No	2: No	2: Limited
Funder ClosestClin 10	1: No	1: No	1: No	1: No	1: Limited

^a^Secondary data on the four background factors, collected from the research funders’ institutional homepages

**Fig. 2:**
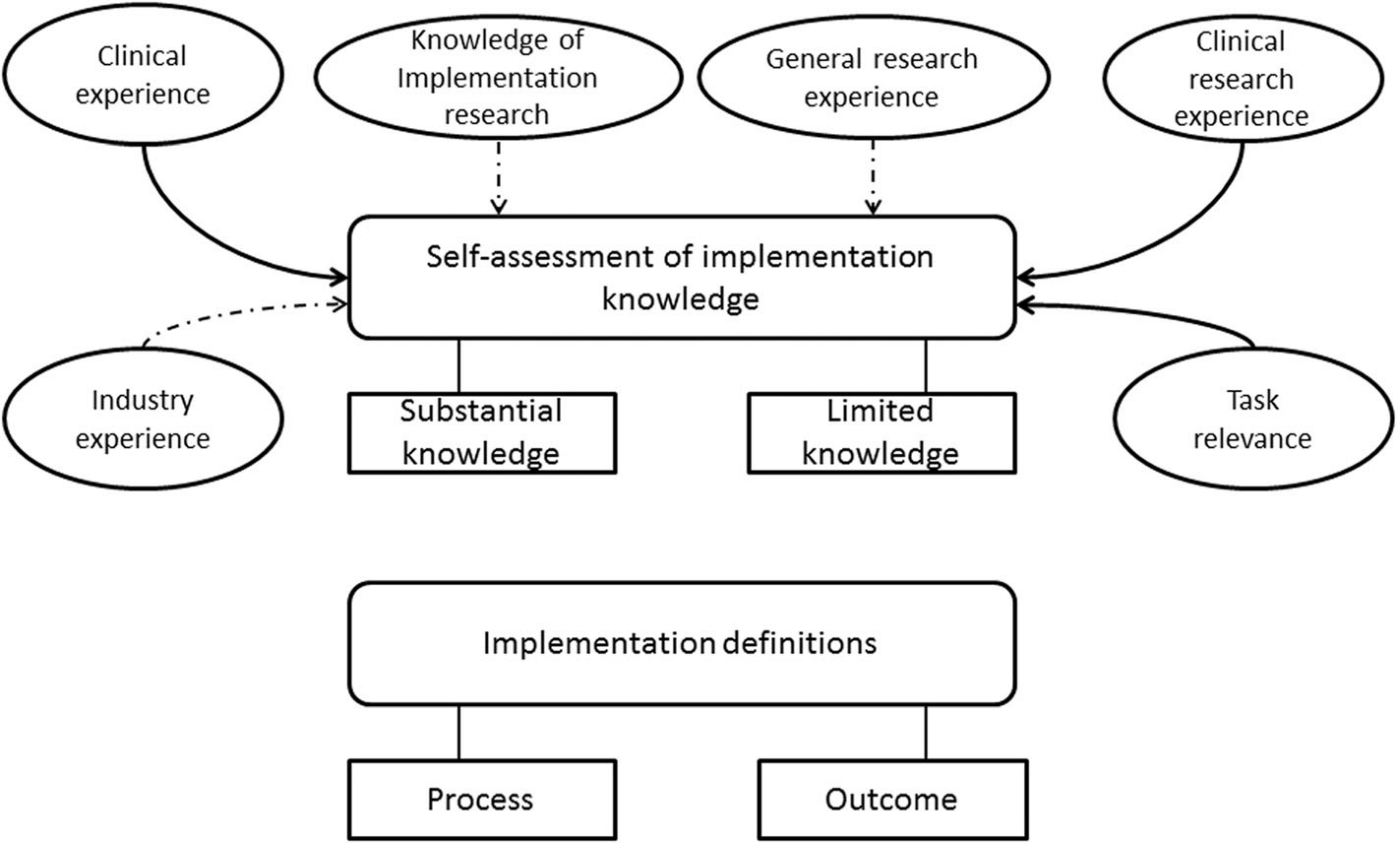
Unpacking research funders’ implementation knowledge. The grounded model emerged from the empirical findings and from relating the empirical findings to existing literature. The grounded model provides a conceptual framework for explaining research funders’ implementation knowledge. Clinical experience, clinical research experience and task relevance were clearly connected to self-assessment of implementation knowledge, which are indicated by bold arrows. Several factors were not clearly connected to self-assessment of implementation knowledge, namely industry experience, knowledge of implementation research and general research experience, which are indicated by dashed arrows. No connection was found between implementation definitions and self-assessment of implementation knowledge

Based on the empirical findings, ‘clinical research experience’, ‘clinical experience’ and ‘task relevance’ are the factors with the strongest influence on self-assessed implementation knowledge, whereas the remaining factors (i.e. general research experience, industry experience and knowledge of implementation research) have a more limited influence. This is illustrated in Fig. 2, which depicts the grounded model. The following section explains the factors in the grounded model and compares our findings with those from previous research to establish the grounded model in relation to existing literature.

## Discussion

This study aimed to explore the nature of research funders’ implementation knowledge by studying their definitions of ‘implementation’ and their self-assessed implementation knowledge, and by identifying the factors that influence their self-assessed implementation knowledge. Figure 2 shows the grounded model that emerged (1) from the empirical findings and (2) from relating the empirical findings to existing literature. Below, we describe the components of the grounded model and discuss them with reference to existing literature.

Previous research about policy-makers’ use of research findings identifies inadequate awareness of research findings as a barrier [52, 63–65], while awareness is a facilitator [64, 66, 67]. Similarly, knowledge of something has been identified as a barrier when it is lacking [52, 63–65, 68] or as a facilitator when present [64, 66, 67]. Although most research funders in our sample lacked awareness and explicit knowledge of implementation research, the majority defined implementation as a process, while a minority perceived it as an outcome.

Research funders’ ‘implementation’ definitions describe one aspect of their implementation knowledge. Whether ‘implementation’ is defined as a process or an outcome entails consequences for research funders’ facilitative roles. For instance, if research funders enact facilitative roles, the number and content of these roles may vary greatly, depending on their implementation definitions. If implementation is defined as a process, several facilitative roles before, during and after implementation are relevant since there is a continuum of activities that take place over a long period. In contrast, if implementation is defined as an outcome, there may be only a few roles in the healthcare context that are relevant for a research funder (e.g. the role of checking the degree of implementation). The distinction between implementation as an outcome and as a process is also made in implementation research, where researchers have depicted implementation as a process [6, 38, 42–46]. Defining implementation only as an outcome would mean viewing implementation as fairly uncomplicated, in contrast to the picture emerging from some 40 years’ implementation research showing that it is a highly complicated process [14, 69, 70]. Moreover, while in principle a single research funder might define ‘implementation’ both as an outcome and as a process, we observed no such instances in our data. Instead, we observed a strong tendency among research funders to emphasise either process or outcome aspects when they define ‘implementation’.

In terms of funding levels, there were only minor differences among the three types of funders. A majority (FarBas and ClosestClin) or at least half (CloserBoth) of the research funders adhered to a process view. However, the implementation definitions did not, in general, cover the second main insight from implementation research – that implementation requires a strategy for identifying and addressing barriers to implementation, as well as facilitators thereof [28, 45, 47, 71]. Acknowledging that implementation requires a process is a good starting point, but in order to appreciate the complexity of implementation, one needs to be aware of that implementation requires a strategy to address barriers to and facilitators of implementation. Without these insights, the research funders do not possess a complete picture of implementation and thus successfully performing roles that go beyond evaluating and funding grant proposals becomes difficult. Further, knowledge of implementation research was not acknowledged as an important factor contributing to implementation knowledge (Table 2). Our data thus indicate a weak link between this factor and self-assessed implementation knowledge (dashed arrow in Fig. 2).

Research experience was of two different types – general research experience and clinical research experience. Research funders explicitly stated their view that clinical research experience contributes to substantial self-assessed implementation knowledge, but a lack of clinical research experience was not explicitly raised as being among the factors contributing to limited self-assessed implementation knowledge. Nevertheless, a clear connection emerged between lack of clinical research experience and limited self-assessed implementation knowledge, as Tables 2 and 3 show. Consequently, we depict a clear connection between self-assessed implementation knowledge and clinical research experience (bold arrow in Fig. 2). However, only CloserBoth research funders raised clinical research experience as an important factor contributing to their self-assessed implementation knowledge; in general, too, only they possessed clinical research experience (Tables 2 and 3). This finding is in line with our sampling assumptions, namely, we had assumed that funders close to implementation contexts and engaged in funding clinical research would display higher levels of experience-based implementation knowledge. On the other hand, the ClosestClin research funders, who are closest to implementation contexts and fund primarily clinical research, neither cited clinical research experience as a factor contributing to their self-assessed implementation knowledge nor possessed clinical research experience (Tables 2 and 3), and these findings contradict our sampling assumptions. Accordingly, operating and being organisationally close to the theatre of implementation, i.e. the healthcare system, does not necessarily provide such research funders with much implementation knowledge. Indeed, the findings of this study imply that experience-based knowledge, such as clinical research experience, contribute to implementation knowledge and thus lack of such experience could lead to limited implementation knowledge. The research funding organisations could balance out this lack of experience-based knowledge by providing, for instance, training in implementation science but this is apparently not the case with ClosestClin funding organisations.

On the other hand, for general research experience, the connection with self-assessed implementation knowledge is less clear-cut than for clinical research experience. General research experience was considered a basis for limited implementation knowledge by only a few research funders (Table 2), indicating that these two parameters (i.e. general research experience and implementation knowledge) were perceived as completely distinct from each other. Comparing the three funding levels, a lack of general research experience appears to be connected to limited self-assessed implementation knowledge, but possessing general research experience is not connected to substantial self-assessed implementation knowledge (Table 3). Consequently, the connection between general research experience and self-assessed implementation knowledge is only tenuous (dashed arrow in Fig. 2). Most of the research funders possessed general research experience and the only group deviating from this pattern were the ClosestClin research funders, most of whom lacked general research experience (Table 3).

Previous studies have made no explicit distinction between general and clinical research experience in terms of policy-makers’ decisions [53, 54, 72]. Our findings therefore shed light on this distinction, which may be important in the context of healthcare. There, clinical research (e.g. the study of new methods for chronic disease self-management [73]) may be assumed to be more relevant, from an implementation point of view, than general research experience obtained in a laboratory setting (e.g. basic research on how voluntary exercise affects mouse behaviour [74]). Consequently, conducting research in the healthcare setting, i.e. the actual implementation context, should boost research funders’ implementation knowledge more than doing research in a laboratory, detached from the implementation context. In line with this assumption, we found a clear connection between clinical research experience and self-assessed implementation knowledge, whereas the connection that emerged between general research experience and self-assessed implementation knowledge was relatively weak (bold and dashed arrows respectively in Fig. 2).

Research funders’ previous practical experience was manifested in two ways – clinical experience and industry experience. According to many research funders, clinical experience contributed to their substantial self-assessed implementation knowledge. Lack of clinical experience was not, on the other hand, explicitly mentioned as a factor contributing to limited self-assessed implementation knowledge (Table 2). However, a comparison across the three funding levels showed that clinical experience was in many cases connected to substantial self-assessed implementation knowledge, whereas its absence was connected to limited self-assessed implementation knowledge (Table 3). Consequently, in general, the findings from the interviews support those from the secondary data and vice versa – together, they indicate a clear connection between clinical experience and self-assessed implementation knowledge (bold arrow in Fig. 2). Only the CloserBoth research funders raised clinical experience as an important factor contributing to their self-assessed implementation knowledge, and in general possessed clinical experience (Tables 2 and 3). Again, this was in line with our sampling assumptions concerning closeness to implementation contexts and the type of research funded. Instead, the research funders operating closest to implementation (ClosestClin) did not, in general, raise clinical experience as an important factor contributing to their self-assessed implementation knowledge (Table 2). However, based on the secondary data, there were some research funders (ClosestClin) who possessed clinical experience, but in these cases, clinical experience corresponded to limited self-assessed implementation knowledge (Table 3). Additionally, these findings show that an experience-based factor – clinical experience – could be a contributing factor to increased implementation knowledge and that the experience-based factors are connected to the individuals rather than their funding organisations. As mentioned previously, this kind of lack of experience could be compensated by training in implementation science.

Previous studies have paid scant attention to the connection between policy-makers’ use of evidence and their clinical experience. Oliver et al. [54], in their extensive review of studies focusing on policy-makers’ use of research results, identify only two studies in this area [75, 76], neither of which explicitly acknowledges the role of policy-makers’ clinical background. Our findings that clinical experience strongly contributes to policy-makers’ substantial self-assessed knowledge and that its lack contributes to a limited self-assessed knowledge is a first attempt to address this gap. Industry experience, in turn, has not been identified in previous research among the factors hindering or facilitating policy-makers’ use of evidence, and was cited by only a few research funders as a factor contributing to their self-assessed implementation knowledge (Table 2). Moreover, in general, the research funders lacked this type of professional background (Table 3) and, accordingly, this connection in our model is only tentative (dashed arrow in Fig. 2).

Besides various sources of knowledge, we identified an additional factor that influences self-assessed implementation knowledge – ‘task relevance’. Research funders who perceived that implementation was part of their tasks had either substantial knowledge or, alternatively, an ambition to improve their limited knowledge (CloserBoth and ClosestClin funders in Table 2). In contrast, research funders (FarBas funders in Table 2) who perceived that implementation was not part of their tasks had limited knowledge and did not even consider this a problem. This finding is supported by previous studies on planned behaviour, where people’s intentions to act were found to depend on their attitudes toward the tasks concerned and whether they perceived them as relevant [77, 78]. When applied to research funders, the perception of a facilitative role as relevant is connected to a higher probability of a given action, such as acquiring implementation knowledge, and vice versa. Task relevance was found to be connected to both limited (when it is lacking) and substantial (when present) self-assessed implementation knowledge. It thus constitutes a clear factor (bold arrow in Fig. 2) that is also supported by the theory of planned behaviour.

Finally, we considered the connections between implementation definitions and self-assessed implementation knowledge. As Table 2 shows, respondents may have an outcome view of implementation irrespective of their level of self-assessed implementation knowledge, and this is true of the process view (‘missing’ arrow in Fig. 2) as well. Overall, our interpretation is that the experience-based implementation knowledge possessed by research funders is comparable to some of the findings from implementation research because a majority of the research funders perceived implementation as a process. Nevertheless, the overall level of self-assessed implementation knowledge was considered limited, which may also explain why research funders’ lack knowledge concerning certain aspects of the implementation process such as identification of barriers to and facilitators of behavioural change [79].

The grounded model may be seen as complementing more general implementation models and frameworks, which provide guidance on general factors that either hinder or facilitate implementation [28, 45, 80]. In fact, while existing implementation models focus on the implementation context in healthcare, laying great emphasis on practitioners (e.g. attitudes), patients (e.g. adherence to treatment) and organisational factors (e.g. resources) [27, 40, 43, 44, 47, 81], they leave research funders in the background. However, in certain situations, the funders can facilitate the steps leading to implementation, and the grounded model in Fig. 2 provides a conceptual framework for explaining funders’ implementation knowledge. For instance, research funders need implementation knowledge to stimulate cooperation between researchers and users or to make decisions about funding for implementation [15, 22, 25, 29]. Such actions, combined with appropriate implementation knowledge, may diminish the knowledge–practice gap.

At a more theoretical level, Fig. 2 provides a middle-range model that explains individual actors’ knowledge by relying on knowledge that can be acquired both through practical experience and by consulting research findings [82]. However, most of the conceptual model’s factors influencing self-assessed implementation knowledge are related to individuals’ lifelong experience, and are difficult, if not impossible, to change through a behavioural change intervention. This is true, for instance, for ‘lack of clinical experience’. The conceptual model contributes to the literature on policy-makers’ use of research evidence by emphasising that there are important factors that can explain policy-makers’ implementation knowledge and that go beyond the actual research evidence and its official sources. Our model particularly stresses the relevance of experience-based knowledge contributing to policy-makers’ implementation knowledge. Furthermore, one of our sampling assumptions was that research funders’ closeness to implementation context would provide them with experience-based implementation knowledge. In contrast, our findings imply that research funders’ implementation knowledge is not dependent on their funding organisation but rather on their individual experiences.

Previous policy research has focused mainly on science-based knowledge [53, 54], leaving relatively unexplored the alternative sources of knowledge that influence policy-making [83]. One exception is Oliver et al. [83], who surveyed a sample of public-health policy-makers concerning the sources of information they used in policy-making. They found that government scientific databases, such as the United Kingdom’s National Institute for Health and Care Excellence, were the main information sources, along with personal contacts with middle managers. Consequently, Oliver et al.’s [83] findings illustrate the relevance of science-based knowledge among health policy-makers but do not identify the relevance of experience-based knowledge, which we found in our study to be the most important aspect. Our model illustrates that health research funders are a category of policy-makers who, with their strategic position between practice and research, may possess implementation knowledge comparable to some important scientific findings.

However, the fact that these research funders are a category of policy-makers who work at the interface between research and practice makes them special but not unique. Hospital policy-makers, health ministries and education policy-makers, too, work between research and practice. Since experience-based factors were found to be pivotal in constituting research funders’ knowledge, this may point to a need to adjust existing policy-maker models and evaluate whether similar results concern other categories of policy-makers as well.

### Limitations and future research

An inductive approach was employed to study research funders’ implementation knowledge and, because our understanding of this topic is largely incomplete, semi-structured interviews were used to explore it. To enhance the relevance of the findings and strengthen the grounded model, a systematic coding procedure was adopted that allowed the codes to be based on quotations from the respondents. To offset the potential limitation of subjectivity in our coding, an inter-rater coding procedure was employed and the coding adjusted accordingly.

Another limitation of this study is that it is based on a restricted number of Swedish research funders. They were purposefully selected as being representative of a wide range of funding organisations in terms of closeness to implementation and the types of research funded, but as stated earlier, the research funders possessed experience-based knowledge, which was not clearly connected to closeness to implementation or the type of research funded by their organisations. Consequently, this study showed that it is the individuals’ experiences that are more relevant in forming implementation knowledge than their organisational affiliation. These findings could apply even to other countries and contexts, for instance, experience-based implementation knowledge could be universal. Future studies could be conducted on large samples of research funders as well as of other kinds of policy-makers in various countries to test the findings of this study. Moreover, studies that also assess the implementation knowledge of implementers and clinical decision-makers could be set up. Such future studies would expand our understanding of the impact of, for instance, implementation research on all the actors involved in implementation. Finally, the number of respondents interviewed and included in this study is relatively small (*n* = 18), which means that the results and the connections highlighted in Fig. 2 should be considered tentative, and thus need to be tested with a larger sample. However, the secondary data from the funders’ institutional homepages did not contradict but, rather, confirmed the interview data, making the overall findings more credible [59].

## Conclusions

Even if our study focuses on a high-income country (Sweden), according to our knowledge, there have been no previous studies, neither in low- or high-income contexts, investigating the implementation knowledge of policy-makers such as research funders. Our study is accordingly the first initiative in this area. Our findings point to the need to inform policy-makers about some of the findings from implementation research, but simultaneously demonstrate that implementation knowledge acquired through professional experience (e.g. clinical research, general research or clinical experience) may provide experience-based implementation knowledge. Further, research funders’ self-assessed implementation knowledge may increase if they perceive that implementation is a relevant task for them. On the other hand, implementation researchers should be concerned because only a few of the research funders studied expressed explicit knowledge of implementation research.

These findings have three important implications. First, professional experience can provide implementation knowledge but does not provide a complete picture of implementation and, in cases where the research funders do not have experience of the factors (i.e. clinical experience) that contribute to implementation knowledge, the research funding organisations could provide training in implementation science. Second, implementation researchers need to employ various strategies to reach policy-makers and explain the usefulness of findings from implementation research since, if these findings are not used, their relevance may be questioned. Third, inducing research funders to embrace implementation as part of their tasks may boost their self-assessed implementation knowledge. These implications relate both to research funders in suggesting how they can evaluate and expand their knowledge of implementation, and to implementation researchers, in indicating a need to identify more appropriate forms and channels for communicating their findings to policy-makers.

## Additional file

Additional file 1: Structured overview of implementation definitions and self-assessment of implementation knowledge grounded on quotes (DOCX 40 kb)

## Data Availability

The data supporting the conclusions of this article are included within the article (and its additional file).
