# Supplementary material for: An inductive exploration of the implementation knowledge of research funders
Source: Health Res Policy Syst. 2019 Jul 18;17:67. doi: 10.1186/s12961-019-0472-8 (PMC6637601; doi:10.1186/s12961-019-0472-8)
Supplement: Supplementary file 1 — Structured overview of implementation definitions and self-assessment of implementation knowledge grounded on quotes (DOCX 40 kb) [file 12961_2019_472_MOESM1_ESM.docx]

| **Additional file 1**  **Structured overview of implementation definitions and**  **self-assessment of implementation knowledge grounded on quotes** | |  | |
| --- | --- | --- | --- |
| **First-order categories** | **Second-order themes and representative quotes** | **Aggregate dimensions** | |
| **IMPLEMENTATION DEFINITIONS** | | | |
| A. A process that leads to guidelines which then are used | **Use of guidelines**  A1. “[Y]ou then get these results applied and that you follow up their application and you write guidelines. Yes, when one has written the guidelines some people at the clinic become responsible and they will follow up guidelines. You can’t do anything else. I mean, today we work on the basis of guidelines, so everybody knows how to work with the guidelines.” (Respondent 1 – CloserBoth Funder 5)  A2. “[T]hat it goes from research conclusion, based on one or several publications, to clinical guidelines and so that it also becomes that way in practice, and one can check this. One starts to check this from the quality databases where one can actually see if that patient was treated according to guidelines.” (Respondent 2 – CloserBoth Funder 6) | |  |
| B. Utilization of research knowledge in practice | **Research findings are used in practice**  B1. I define it as the new research findings being used – utilized and used in practice.” (Respondent 2 – FarBas Funder 1)  B2. ”[I]t is utilized in the practice where it is assumed to be utilized. Yes, that knowledge is used in practice. This is put very simply.” (Respondent 1 – CloserBoth Funder 4) | | Outcome view |
| C. The individual healthcare professional updates himself and changes her practice | C1. “[T]he individual head of operations and chief physicians change their practice. So if people don’t care to update themselves that one should not use leeches in medical practice there is nothing we can do…but in healthcare, I would still claim, that we have a driving force and we don’t want to do wrong.” (Respondent 1 – ClosestClin Funder 7) | |  |
|  | **Simple process of introducing new research results in practice** | |  |
| D. Make sure that research results are introduced in healthcare | D1. “[M]ake sure that a product or a service or a process starts and works in real life.” (Respondent 1 – FarBas Funder 1)  D2. “A real implementation is to make sure that results are used.” (Respondent 1 – FarBas Funder 2) | |  |
| E. One introduces new methods based on research | E1. “Introduction of a new method or a device” (Respondent 2 – FarBas Funder 2)  E2. ” [T]hat it is integrated with the practical work in healthcare. One can understand that it is not an easy task.” (Respondent 1 – ClosestClin Funder 8) | |  |
| F. A process requiring adaptation of activities | **Complex process of translating research results to practice**  F1.”So now comes the question whether I know what I’m talking about. For me implementation is that one plainly translates a research result to healthcare… And to do this, I think, one needs even here a little research process, I mean some kind of implementation research, good follow up and so on. It is not only about talk to talk, rather one need to know all the time what one is doing.” (Respondent 1 – CloserBoth Funder 6) | |  |
|  | F2. “It is completely illogical. You can’t foresee it. There are some general steps. You need to be flexible – to be able to adjust, you need to have a plan, you need to have the right people on board, and you need to know which steps you need to go through. And then you need to have an adaptive project plan that can be adjusted, depending on the reality you find when you approach the goal.” (Respondent 1 – FarBas Funder 3) | | Process view |
| G. A process including different steps where the goal is changed behavior | G1. “[T]o transfer research result to clinical practice…what it means for organization and which resources are required when one changes healthcare, whether it is a new method or a new farmacology. I have great experience of how that process is conducted.” (Respondent 2 – CloserBoth Funder 5)  G2. “[T]he goal of implementation is changed behavior so that one gets another outcome for the customer – the patient. And it can be more or less difficult, depending on what’s going to be implemented… And then it has a lot to do with education – motivating, setting clear goals, arranging activities, carrying them out, following up and evaluating. So it’s sort of like that: A lot of support is often needed.” (Respondent 2 – ClosestClin Funder 8) | |  |
| **SELF-ASSESSMENT OF IMPLEMENTATION KNOWLEDGE** | | | |
| A. I don’t have any detailed knowledge | **Very little knowledge**  A1. “Not much at all. You mean in healthcare? No.” (Respondent 1 – ClosestClin Funder 8)  A2. “No, not that many. I’m a researcher so we don’t work that much with implementation here. No, I don’t have any extensive knowledge about that.” (Respondent 1 – FarBas Funder 2) | |  |
| B. I’ve heard about that | B1. “They are very rudimentary. I’m an experimental person.” (Respondent 1 – CloserBoth Funder 6) | | Limited knowledge |
|  | B2. ”They are very superficial. I’ve heard about that and so.” (Respondent 1 – ClosestClin Funder 9) | |  |

| **Additional file 1**  **Continued** | |  | |
| --- | --- | --- | --- |
| **First-order categories** | **Second-order themes and representative quotes** | **Aggregate dimensions** | |
| C. Little expert knowledge but I can understand that I need to employ them who can this | **Some knowledge**  C1. “Very modest, I mean very little expert knowledge, which doesn’t bother me at all, but I can understand the value of implementation and understand, when we talk about implementation, that I need to employ those who care about this [implementation]. It [possessing implementation knowledge] is kind of not my job.” (Respondent 2 – CloserBoth Funder 4)  C2. ”[W]e work quite a bit with implementation here in this division…it is a question of quite difficult processes, especially when it is about overarching processes that are going to be implemented in the whole county council…I can’t say that I’m expert in implementation but we have knowledge in this division.” (Respondent 2 – ClosestClin Funder 8) | |  |
| D. I’m interested and receive knowledge from people who can implement | D1. “Too little, I dare to say… what is still most exciting is when we talk to different companies that are trying to implement new drugs, new methods and similar things. We talk a lot about that, which is exciting and interesting. I get more of this kind of knowledge from them than I get from the county council’s own healthcare organization.” (Respondent 2 – ClosestClin Funder 9)  D2. ”I’m not a researcher but I’ve been interested about implementation so I’ve read quite a bit and I have contact with researchers…I’ve been very interested of this and would like to create a professorship in the subject.” (Respondent 1 – ClosestClin Funder 10) | |  |
| E. I’m interested to acquire knowledge – read journals but I don’t know what I’m looking for | E1. “I can’t say that I have any specific knowledge… I’m interested in acquiring knowledge. I read journals but I don’t know what I’m looking for.” (Respondent 2 – FarBas Funder 1) | | Limited knowledge |
| F. I’m just a healthcare pragmatist | F1. ”[I have] layman knowledge in a way that we need to absorb and implement, it is part of the physician job according to me…There is not one thing that is the same, I mean this a weird question for us doctors because we need to change all the time.” (Respondent 1 – ClosestClin Funder 7)  F2. ”I’m just a simple pragmatist…I don’t have any specific knowledge about that. I’ve worked a long time in the field, partly I’ve worked in the drug industry…The task was to sell drugs and that is to implement them in healthcare…Then I have also experience from healthcare so I’ve seen how different it is…I’ve been the hospital director at [one large] university hospital.” (Respondent 1 – CloserBoth Funder 4) | |  |
| G. I’ve been working with clinical research about 30 years so I know how to implement | **A lot of knowledge from a healthcare point of view**  G1. “[Y]ou know, if you’ve been involved like I have, you get experience. I’ve been working in clinical research since 1970 so I know. Experience from these years gives knowledge, so to speak. Research results and how to implement them, what’s possible and what isn’t.” (Respondent 1 – CloserBoth Funder 5)  G2. ”I have quite profound…I’ve been working with clinical studies as one part of my research during 25 years or something like that and I’m co-responsible for the Nordic care program for my disease and we’ve implemented a lot of results all the time…So I have a very extensive experience of implementing research results in practice.” (Respondent 2 – CloserBoth Funder 6) | |  |
| H. I’m responsible for a lot of implementation in my healthcare region so I know much about implementation | H1. ”I have quite lot – I’m responsible for a lot of implementation in healthcare in this region. So I can say that I know that much about implementation of, and not only about medical technology, but also prevention and public health work and so on. It’s not a physician deed.” (Respondent 2 – FarBas Funder 2)  H2. “Yes, a lot [of knowledge]. I’ve been a director of [a clinical unit] for many years and I’ve also been the director of [a specialist medical research unit], so I have quite extensive experience of what it means, organizationally and from a resource point of view, when you change healthcare. Whether it’s a new method or a new drug, I have extensive experience of what that process is like.” (Respondent 2 – CloserBoth Funder 5) | | Substantial knowledge |
| I. I’ve been a director of R&D in industry so I have good knowledge | **A lot of knowledge from an industry point of view**  I1. “I’ve been a director of R&D at [a large multinational company] for [several] years, so I have good knowledge about that.” (Respondent 1 – FarBas Funder 3) | |  |
|  | | | |
|  |  | |  |
